# National trends in the prevalence of glycemic control among patients with type 2 diabetes receiving continuous care in Thailand from 2011 to 2018

**DOI:** 10.1038/s41598-021-93733-4

**Published:** 2021-07-12

**Authors:** Boonsub Sakboonyarat, Warabhorn Pima, Channarong Chokbumrungsuk, Taksin Pimpak, Sirikorn Khunsri, Supak Ukritchon, Worarachanee Imjaijitt, Mathirut Mungthin, Wisit Kaewput, Bhophkrit Bhopdhornangkul, Nattapol Sathavarodom, Pyatat Tatsanavivat, Ram Rangsin

**Affiliations:** 1 Department of Military and Community Medicine, Phramongkutklao College of Medicine, Bangkok, Thailand; 2 Medical Research Network of the Consortium of Thai Medical Schools, Bangkok, Thailand; 3 National Health Security Office, Bangkok, Thailand; 4 Office of Research and Development, Phramongkutklao College of Medicine, Bangkok, Thailand; 5 Department of Pharmacology, Phramongkutklao College of Medicine, Bangkok, Thailand; 6 Department of Microbiology, Phramongkutklao College of Medicine, Bangkok, Thailand; 7 Division of Endocrinology, Department of Medicine, Phramongkutklao Hospital, Bangkok, Thailand; 8 Department of Medicine, Faculty of Medicine, Khon Kaen University, Khon Kaen, Thailand

**Keywords:** Type 2 diabetes, Risk factors, Epidemiology

## Abstract

Diabetes is one of the largest global health problems and exhibits a constantly increasing trend. A series of nationwide hospital-based cross-sectional surveys of clinical outcomes was performed annually from 2011 to 2015 and 2018 among patients with type 2 diabetes aged ≥ 20 years receiving medical care for at least 12 months. A two-stage stratified cluster that was proportional to the size sampling technique was used to select a nationally and provincially representative sample of patients with type 2 diabetes in Thailand. A total of 186,010 patients with type 2 diabetes were enrolled in the study from 2011 to 2018. The prevalence of adequate glycemic control (hemoglobinA1c level < 7.0%) among patients with type 2 diabetes were estimated to be 34.5% (95%CI 33.8–35.2%) in 2011, 33.0% (95%CI 32.4–33.6%) in 2012, 34.7% (95%CI 34.1–35.4%) in 2013, 35.5 (95%CI 34.9–36.1%) in 2014, 35.6 (95%CI 35.0–36.2%) in 2015, and 35.6% (95%CI 35.0–36.2%) in 2018, respectively (*p* for trend < 0.001). Independent factors related to poor glycemic control (hemoglobinA1c ≥ 7%) were being female, younger aged, living in the northeastern region, received care form hospitals lower than regional level, under universal health coverage scheme, greater duration of diabetes, higher body mass index level and absence of hypertension comorbidity.

## Introduction

Currently, diabetes is one of the largest global health problems and exhibits a constantly increasing trend. The International Diabetes Federation has estimated that 450 million people are living with diabetes, and estimates this figure will reach 642 million in 2040^1^. More than 60% of the world population living with diabetes is from Asian countries^2^. Additionally, Asians may be more susceptible to the development of type 2 diabetes and may have a higher risk of developing diabetes complications than other ethnicities^3^.

In Thailand, the 5th National Health Examination Survey (NHES V) conducted in 2014 found that the prevalence of diabetes among Thais aged over 20 years was 9.9, 8.9 and 10.8% among total adults, males and females, respectively^4^. Nevertheless, the percentage of undiagnosed type 2 diabetes was 51.2 and 41.3% among male and female patients with diabetes, respectively. Additionally, 4.2% of males and 1.7% of females received a diagnosis type 2 diabetes but were untreated. In terms of residence, the prevalence of type 2 diabetes, undiagnosed and controlled among Thai adults in urban and rural areas were comparable^4^. Currently, diabetes is a major health problem in Thailand. The disability-adjusted life years from diabetes in Thailand constituted the 7th leading cause of morbidity among men and the 2nd leading cause among women in 2013^5^. Proper glycemic control will reduce the risk of diabetes complications, particularly microvascular complications^6,7^. Adult diabetes cases benefit from proper glycemic control, indicated by glycosylated hemoglobin (HbA1c) levels < 7% (53 mmol/mol)^8^. The HbA1c level was adopted as the assessment method for glycemic control by the Diabetes Clinical Practice Guidelines from the National Health Security Office (NHSO), Thailand, in 2011^9^. The indicated target for the attainment of glycemic control is an HbA1c value < 7% (53 mmol/mol) in nonpregnant adult diabetes cases.

In 2015, many countries worldwide began to move toward providing universal health coverage based on the Sustainable Development Goals. In Thailand, universal health coverage was implemented in 2002^10^ and included free services for all basic health problems, including type 2 diabetes. The present study comprised an annual national study among patients with type 2 diabetes aiming to determine national trends in the prevalence of glycemic control among patients with type 2 diabetes receiving continuous care in Thailand from 2011 to 2018 which was a decade after implementing the universal health coverage scheme in Thailand. Furthermore, we identified factors associated with poor achievement of glycemic control.

## Methods

### Study design and participants

**Figure 1:**
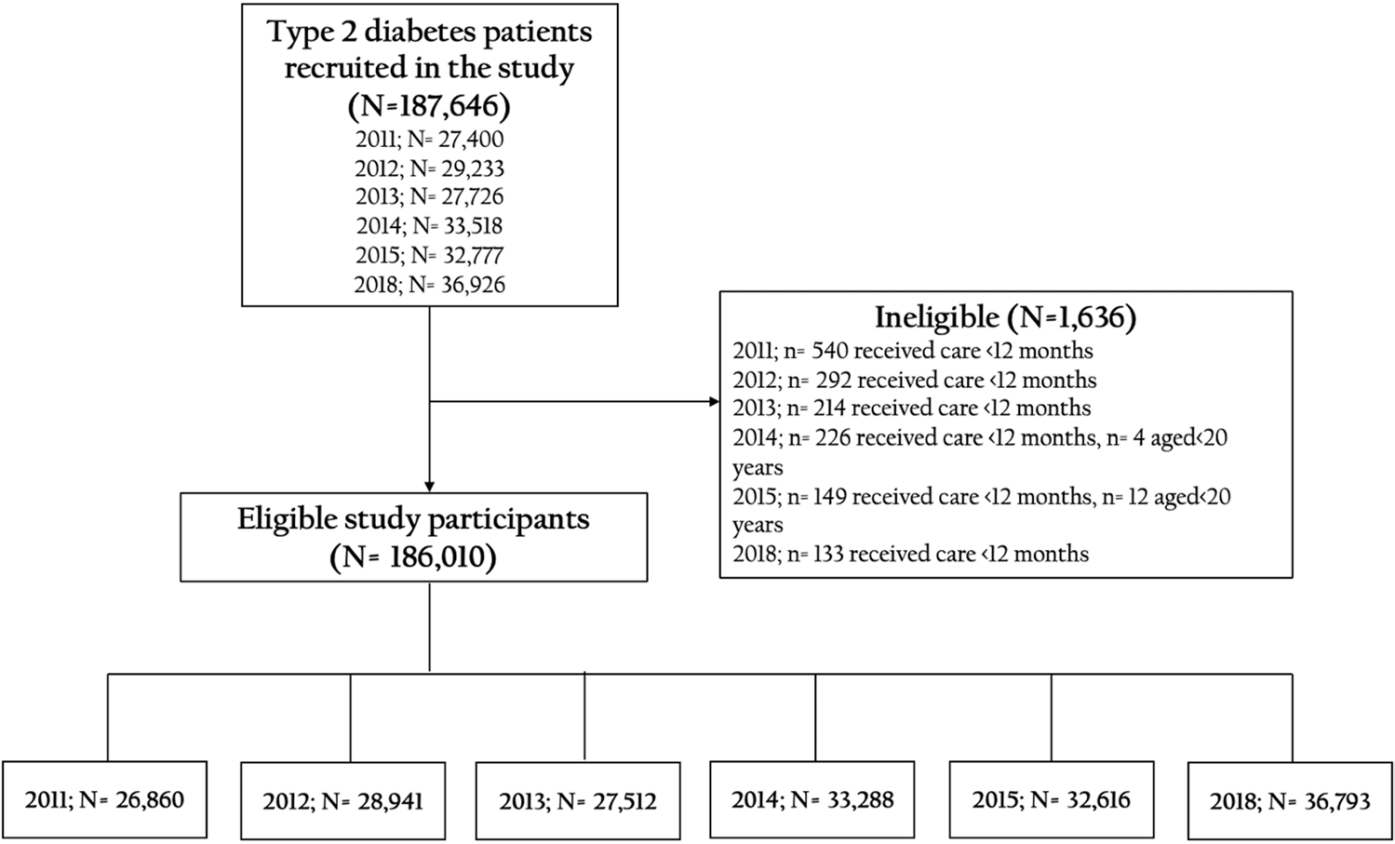
Flow of enrolled patients with type 2 diabetes receiving continuous care in Thailand from 2011 to 2015 and 2018.

A series of annual cross-sectional studies was performed from 2011 to 2015, and 2018 to evaluate the status of diabetes care among patients with type 2 diabetes attending public hospitals of the Ministry of Public Health (MoPH) nationwide in Thailand. The participating hospitals also included public hospitals and private clinics in the Bangkok Metropolitan Region supported by Thailand’s NHSO program. The inclusion criteria comprised patients with type 2 diabetes aged 20 years in 2011, 2012, 2014, 2015 and 2018 studies and aged 35 years in the 2013 study and older who visited clinics and received medical care in the targeted hospitals for at least 12 months (Fig. 1). Patients participating in any other clinical trials were excluded.

A stratified two-stage cluster sampling proportional to the size method was used to select a nationally and provincially representative sample of patients with type 2 diabetes in Thailand. For each province outside the Bangkok Metropolitan Region, the hospital universe is defined by all hospitals that are public hospitals under the MoPH. For the Bangkok Metropolitan Region, the hospital universe is defined as all hospitals and clinics that participate in Thailand’s NHSO program. The study sample was a stratified sample drawn from a subset of all MoPH hospitals in Thailand, including all public and private clinics in the Bangkok Metropolitan Region under the NHSO program. Hospitals were stratified in two levels. The first level was the province, which constituted 77 strata (from the 77 provinces in Thailand), and the second level was the hospitals in each province. The hospitals in each province were stratified in 5 strata based on their sizes as follows: regional center hospital (> 500 beds), provincial general hospital (200–500 beds), large community hospital (90–120 beds), medium-sized community hospital (60 beds) and small community hospital (10–30 beds). The primary sampling unit was the hospital. After each study site received the assigned sample size of patients with type 2 diabetes, the study site’s coordinator calculated the quota of study samples for every clinic that provided medical care for patients with type 2 diabetes in each participating hospital. In each selected hospital, a patient with type 2 diabetes provided type 2 diabetes care by the particular hospital for more than 12 months and attended the clinic on the enrollment date was invited to participate in the study. This process continued until the assigned number of consecutive patients with type 2 diabetes for that clinic was reached. Not all university hospitals were included in the study.

### Data collection

At each clinic, health care personnel (usually a registered nurse) invited patients with a pre-existing diagnosis of type 2 diabetes in a consecutive sequence to participate in the study. The patients were asked to sign a consent form to allow the investigators to review and abstract their medical records. Data abstracted from the patients’ medical records included baseline information, status of diabetes complications, the results of different individual laboratory tests and medications prescribed to control diabetes. A standardized case report form (CRF) was used for medical record abstractions (Supplemental file). The CRF was completed by well-trained registered nurse using a standard protocol and was sent to the Medical Research Network of the Consortium of Thai Medical Schools (MedResNet) central data management unit in Nonthaburi, Thailand. For transferring the CRF to electronic dataset, an automated scan to database software solution was used to extracts data and converts them to database records.

### Ethics consideration

The study was approved by the Ethics Review Committee for Research in Human Subjects, Thailand Ministry of Public Health, the Royal Thai Army Medical Department Ethics Review Board, and the local institutional review boards of the local participating hospitals. Written informed consent was obtained from the participants after they read the information sheet and signed the consent form. The participants consented agreeing the WMA Declaration of Helsinki–Ethical principles for medical research involving human subjects.

### Statistical analysis

Statistical analyses were performed using StataCorp. 2021. *Stata Statistical Software: Release 17*. College Station, TX: StataCorp LLC USA. The analysis was restricted to the respondents with the sample weighted against the national database for type 2 diabetes populations in 2011, 2012, 2013, 2014, 2015 and 2018. Sample sizes varied according to the primary outcome analyzed. Standard weighting procedures were used to construct sample weights considering the multistage stratified cluster sampling survey scheme^11^. Population weighted numbers, weighted prevalence rates, and odds ratio (OR) were calculated. The prevalence of outcome indicators was described as a percentage with a 95% confidence interval (95% CI). *P* for trend was calculated using *chi*-square statistics. ANOVA was used for statistical analysis to determine differences between the means of the continuous outcomes of interest. Binary logistic regression analysis was used to determine the risk factors for poor control of HbA1c (HbA1c ≥ 7.0%), and the magnitude of association was presented as crude odds ratio (OR) with 95%CI. Multivariate analysis was performed using logistic regression analysis. Adjusted odds ratio (AOR) from multivariate analysis was presented with corresponding 95% CI, and a *p*-value less than 0.05 was considered statistically significant.

## Results

### Demographic characteristics

**Figure 2:**
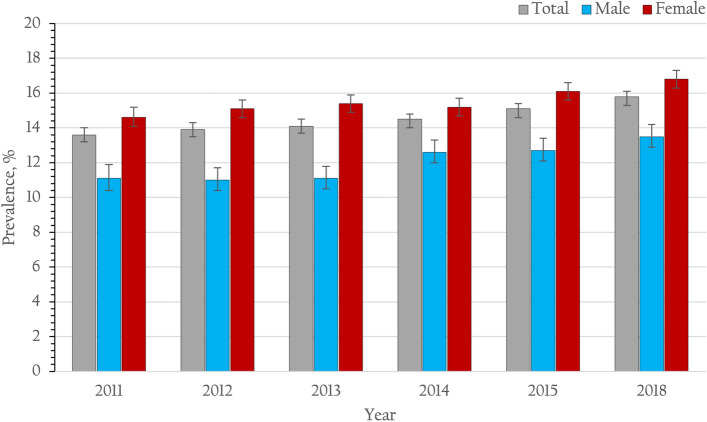
Prevalence of body mass index ≥ 30 kg/m^2^ and 95% CI among patients with type 2 diabetes receiving continuous care in Thailand from 2011 to 2015 and 2018.

From 2011 to 2015, and 2018, a total of 186,010 patients with type 2 diabetes attending the participated clinics were enrolled in the study, including 26,860, 28,941, 27,512, 33,288, 32,616 and 36,793 patients in 2011, 2012, 2013. 2014, 2015 and 2018, respectively. Characteristics of the enrolled patients with type 2 diabetes by survey years are presented in Table1. The mean age of the enrolled patients was 61.1 ± 10.9 years. Approximately one half of the participants were agriculturists. Almost one third (31.5%) of the enrolled patients were male. The majority (74.6%) of the study participants were under the universal health coverage scheme, providing free health care for all Thais. Approximately three fourths (74.4%) of patients with type 2 diabetes had comorbid hypertension. The mean duration of the type 2 diabetes diagnosis was 7.3 ± 4.8 years. One half (50.4%) of the enrolled patients had a BMI ≥ 25 kg/m^2^. Additionally, we observed an increasing trend among the patients with a BMI ≥ 30 kg/m^2^ during the study period (*p* for trend < 0.001) (Table 2 and Fig. 2). The majority (66.2%) of patients were enrolled from community hospitals located at the district level in Thailand. A total of 20.1, 33.5, 32.6, and 13.8% of the patients were located in the north, central, northeast, and south regions, respectively.

**Table 1:** Demographic characteristics of the enrolled patients with type 2 diabetes from 2011 to 2016 and 2018.

Year	Overall	2011	2012	2013	2014	2015	2018	*p*-value
	N = 186,010	N = 26,860	N = 28,941	N = 27,512	N = 33,288	N = 32,616	N = 36,793	
Characteristics	n (%)	n (%)	n (%)	n (%)	n (%)	n (%)	n (%)	
**Gender**								< 0.001^§^
Male	58,600 (31.5)	8017 (29.8)	8777 (30.3)	8586 (31.1)	10,345 (31.1)	10,603 (32.4)	12,272 (33.0)	
Female	127,410 (68.5)	18,843 (70.2)	20,164 (69.7)	18,926 (68.9)	22,943 (68.9)	22,013 (67.6)	24,521 (67.0)	
**Age, years**								< 0.001^§^
20–29	353 (0.19)	73 (0.3)	84 (0.3)	N/A (N/A)	68 (0.2)	63 (0.2)	65 (0.2)	
30–39	4180 (2.3)	776 (2.9)	714 (2.5)	485 (1.8)	796 (2.3)	714 (2.2)	695 (1.7)	
40–49	22,874 (12.3)	3683 (13.8)	4051 (14.2)	3606 (13.2)	4144 (12.4)	3683 (11.2)	3707 (9.9)	
50–59	55,498 (29.8)	8666 (32.3)	9097 (31.4)	8497 (31.0)	9675 (29.1)	9420 (29.0)	10,143 (27.4)	
60–69	61,457 (33.0)	8481 (31.7)	9285 (32.1)	9013 (32.8)	11,028 (33.3)	10,965 (33.9)	12,685 (34.9)	
70–79	33,545 (18.0)	4418 (16.2)	4811 (16.5)	4901 (17.7)	6078 (18.3)	6178 (18.9)	7159 (19.7)	
≥ 80	8103 (4.4)	763 (2.8)	899 (3.0)	1010 (3.6)	1499 (4.5)	1593 (4.6)	2339 (6.3)	
Mean ± SD	61.1 ± 10.9	59.8 ± 10.7	60.0 ± 10.7	60.8 ± 10.5	61.1 ± 11.0	61.5 ± 11.0	62.3 ± 11.0	< 0.001^¶^
**Regions**								< 0.001^§^
Central	62,336 (33.5)	8140 (31.6)	9688 (31.4)	10,432 (37.3)	10,252 (31.4)	11,571 (31.4)	12,253 (31.4)	
North	37,455 (20.1)	5696 (20.8)	5888 (30.6)	3994 (15.3)	6680 (20.6)	6972 (20.6)	8225 (20.6)	
Northeast	60,613 (32.6)	9707 (36.8)	9999 (37.3)	9616 (36.5)	11,783 (37.3)	9444 (37.3)	10,064 (37.3)	
South	25,606 (13.8)	3317 (10.8)	3366 (10.7)	3470 (10.9)	4573 (10.7)	4629 (10.7)	6251 (10.7)	
**Hospital level**								< 0.001^§^
Regional hospital	19,893 (10.7)	3569 (13.6)	3929 (31.4)	4141 (15.8)	2665 (8.2)	2919 (10.1)	2670 (8.0)	
General hospital	42,974 (23.1)	6517 (23.5)	7536 (20.6)	7539 (26.4)	5990 (17.5)	7838 (21.8)	7554 (17.5)	
Community hospital	123,143 (66.2)	16,774 (62.9)	17,476 (37.3)	15,832 (57.8)	24,633 (74.3)	21,859 (68.1)	26,569 (74.5)	
**Health insurance scheme**								< 0.001^§^
Government officer	32,074 (17.2)	4509 (16.4)	5162 (17.7)	4759 (17.0)	5376 (15.8)	5858 (17.4)	6410 (17.4)	
Universal health coverage	138,736 (74.6)	15,351 (57.8)	22,258 (77.1)	21,388 (78.1)	26,245 (79.2)	24,905 (77.2)	28,589 (78.8)	
Social security	7330 (3.9)	845 (3.3)	1187 (4.1)	1131 (4.1)	1327 (4.0)	1335 (3.9)	1505 (3.8)	
Others	7870 (4.2)	6155 (22.5)	334 (1.1)	234 (0.8)	340 (1.0)	518 (1.5)	289 (1.0)	
**Diabetes duration**^**a**^** , years**								< 0.001^§^
< 6^**b**^	90,128 (50.7)	15,087 (59.0)	15,453 (56.3)	14,012 (52.7)	15,626 (46.9)	14,468 (46.2)	15,482 (46.2)	
≥ 6	87,557 (49.3)	10,477 (40.1)	11,981 (43.7)	12,565 (47.3)	17,661 (53.1)	16,861 (53.8)	18,012 (53.8)	
Mean ± SD	7.3 ± 4.8	6.5 ± 4.6	6.81 ± 4.6	7.1 ± 4.8	7.6 ± 4.1	7.8 ± 4.9	7.9 ± 5.3	< 0.001^¶^
**HT comorbidity**								< 0.001^§^
Yes	138,360 (74.4)	18,207 (67.1)	20,370 (69.5)	19,985 (72.1)	25,379 (76.1)	25,520 (77.3)	28,899 (78.1)	
No	47,650 (25.6)	8653 (32.9)	8571 (30.5)	7527 (27.9)	7909 (23.9)	7096 (22.7)	7894 (21.9)	
**Number of antihyperglycemic medication**								< 0.001^§^
None	4821	N/A	N/A	889 (3.2)	1168 (3.5)	1242 (3.8)	1522 (4.1)	
Monotherapy	47,731	N/A	N/A	9783 (35.6)	12,504 (37.6)	12,126 (37.2)	13,318 (36.2)	
Combination therapy	77,657	N/A	N/A	16,840 (61.2)	19,616 (58.9)	19,248 (59.0)	21,953 (59.7)	

SD: standard deviation; HT: hypertension.

^§^Chi-square test, ^¶^ANOVA.

^a^There were 8325 missing values for diabetes duration.

^b^Cut-off points at the median.

**Table 2:** Clinical laboratory test results of the enrolled patients with type 2 diabetes from 2011 to 2015 and 2018.

Year	Overall		2011		2012		2013		2014		2015		2018		*p*-value
	N = 186,010		N = 26,860		N = 28,941		N = 27,512		N = 33,288		N = 32,616		N = 36,793		
Characteristics	n or mean ± SD	% (95% CI) or mean ± SEM for weighted	N or mean ± SD	% (95% CI) or mean ± SEM for weighted	N or mean ± SD	% (95% CI) or mean ± SEM for weighted	N or mean ± SD	% (95% CI) or mean ± SEM for weighted	N or mean ± SD	% (95% CI) or mean ± SEM for weighted	N or mean ± SD	% (95% CI) or mean ± SEM for weighted	N or mean ± SD	% (95% CI) or mean ± SEM for weighted	
**BMI**^**b**^**, kg/m**^**2**^															< 0.001^§^
18.5–22.9	45,424	25.7 (25.5–25.9)	6442	25.9 (25.3–26.4)	6753	25.6 (25.1–26.2)	6675	25.9 (25.4–26.4)	8393	26.2 (25.8–26.7)	8105	25.9 (25.4–26.5)	9056	25.4 (24.9–25.9)	
< 18.5	6557	3.7 (3.6–3.8)	879	3.5 (3.3–3.8)	939	3.6 (3.4–3.8)	893	3.5 (3.3–3.7)	1251	4.0 (3.7–4.2)	1151	3.7 (3.5–4.0)	1444	4.1 (3.9–4.4)	
23.0–24.9	35,610	20.2 (20.0–20.3)	5136	20.7 (20.2–21.2)	5412	20.4 (19.9–20.9)	5245	20.2 (19.7–20.7)	6534	20.3 (19.9–20.8)	6171	19.9 (19.4–20.3)	7112	19.9 (19.4–20.3)	
25.0–29.9	64,249	35.9 (35.7–36.2)	9086	36.3 (35.7–36.9)	9752	36.5 (35.9–37.1)	9477	36.3 (35.7–36.9)	11,273	35.0 (34.4–35.1)	11,322	35.4 (34.8–35.9)	12,719	34.9 (34.3–35.4)	
≥ 30	26,534	14.5 (14.3–14.7)	3426	13.6 (13.2–14.1)	3779	13.9 (13.5–14.4)	3775	14.1 (13.7–14.6)	4675	14.5 (14.1–14.9)	4947	15.1 (14.7–15.5)	5931	15.8 (15.4–16.2)	
Mean ± SD	25.6 ± 4.6	25.5 ± 0.01	25.5 ± 4.4	25.5 ± 0.0	25.6 ± 4.4	25.5 ± 0.0	25.6 ± 4.5	25.5 ± 0.0	25.5 ± 4.6	25.5 ± 0.0	25.7 ± 4.6	25.6 ± 0.0	25.7 ± 4.8	25.6 ± 0.0	< 0.001^¶^
**FPG**															< 0.001^§^
No	18,476	10.9 (10.8–11.1)	4562	17.89 (17.4–18.4)	4211	15.2 (14.7–15.6)	3096	11.6 (11.2–12.0)	2886	8.7 (8.4–9.0)	2221	7.3 (7.0–7.6)	1500	4.93 (4.7–5.2)	
Yes	167,534	89.1 (88.9–89.2)	22,298	82.11 (81.6–82.6)	24,730	84.8 (84.4–85.3)	24,416	88.4 (88.0–88.8)	30,402	91.3 (91.0–91.7)	30,395	92.7 (92.4–93.0)	35,293	95.07 (94.8–95.3)	
**FPG, mg/dL**															< 0.001^§^
< 70	1356	0.8 (0.8–0.9)	199	0.9 (0.8–1.0)	247	1.0 (0.9–1.1)	226	0.9 (0.8–1.0)	243	0.8 (0.7–0.9)	223	0.7 (0.6–0.8)	218	0.6 (0.5–0.7)	
70–130	64,219	38.2 (38.0–38.5)	9072	40.8 (40.1–41.4)	9703	39.0 (38.1–39.7)	9236	37.8 (37.1–38.4)	11,527	37.7 (37.2–28.3)	11,600	37.8 (37.2–38.3)	13,081	36.8 (36.2–37.3)	
≥ 130	101,959	60.9 (60.7–61.2)	13,027	58.3 (57.7–59.0)	14,780	60.0 (59.4–60.1)	14,954	61.3 (60.7–62.0)	18,632	61.5 (60.9–62.0)	18,572	61.5 (61.0–62.1)	21,994	62.6 (62.1–63.2)	
Mean ± SD	153.4 ± 56.1	153.6 ± 0.1	151.0 ± 55.8	151.0 ± 0.4	152.9 ± 57.4	154.0 ± 0.4	154.3 ± 58.0	154.5 ± 0.4	153.7 ± 55.5	153.8 ± 0.3	153.9 ± 55.8	153.9 ± 55.8	154.0 ± 54.7	154.4 ± 0.3	< 0.001^¶^
**HbA1c**															< 0.001^§^
No	41,650	23.2 (23.0–23.4)	7300	27.3 (26.8–27.8)	6814	23.8 (23.3–24.3)	5805	21.2 (20.7–21.7)	7482	23.0 (22.5–23.5)	6309	20.3 (19.8–20.8)	7940	23.6 (23.1–24.1)	
Yes	144,360	76.8 (76.6–77.0)	19,560	72.7 (72.2–73.3)	22,127	76.2 (75.7–76.7)	21,707	78.8 (78.3–79.3)	25,806	77.0 (76.5–77.5)	26,307	79.7 (79.2–80.2)	28,853	76.4 (75.9–76.9)	
**HbA1c, %**															< 0.001^§^
< 7.0	50,777	34.8 (34.6–35.1)	6740	34.5 (33.8–35.2)	7367	33.0 (32.4–33.6)	7610	34.7 (34.1–35.4)	9182	35.5 (34.9–36.1)	9537	35.6 (35.0–36.2)	10,341	35.6 (35.0–36.2)	
7.0–7.9	33,460	23.1 (22.9–23.4)	4518	23.1 (22.5–23.7)	5170	23.3 (22.7–23.9)	4947	22.7 (22.2–23.3)	5894	22.9 (22.3–23.4)	6138	23.5 (22.8–23.9)	6793	23.3 (22.9–24.0)	
8.0–8.9	22,681	15.8 (15.6–16.0)	3100	15.8 (15.3–16.3)	3643	16.5 (16.0–17.0)	3397	15.7 (15.2–16.2)	4031	15.6 (15.1–16.0)	3974	15.3 (14.8–15.8)	4536	15.9 (15.4–16.4)	
9.0–9.9	14,844	10.4 (10.2–10.5)	2095	10.7 (10.3–11.2)	2362	10.8 (10.4–11.2)	2293	10.7 (10.3–11.1)	2616	10.1 (9.7–10.5)	2674	10.2 (9.8–10.6)	2804	9.7 (9.3–10.0)	
≥ 10.0	22,598	15.9 (15.7–16.1)	3107	15.9 (15.4–16.5)	3585	16.5 (16.0–17.0)	3460	16.1 (15.6–16.6)	4083	16.0 (15.5–16.4)	3984	15.5 (15.1–16.0)	4379	15.4 (14.9–15.9)	
Mean ± SD	8.0 ± 2.0	8.0 ± 0.0	8.0 ± 2.0	8.0 ± 0.0	8.1 ± 2.0	8.1 ± 0.0	8.0 ± 2.1	8.03 ± 0.0	8.0 ± 2.1	8.0 ± 0.0	7.9 ± 2.0	8.0 ± 0.0	78.0 ± 2.0	8.0 ± 0.0	< 0.001^¶^
**Lipid test coverage**															< 0.001^§^
No	17,108	9.5 (9.4–9.7)	3252	12.1 (11.7–12.5)	3503	12.0 (11.7–12.4)	2628	9.5 (9.2–9.9)	3167	9.8 (9.4–10.1)	2912	9.0 (8.7–9.3)	1646	4.8 (4.6–5.1)	
Incomplete	27,031	15.2 (15.0–15.4)	4703	18.1 (17.7–18.6)	4389	15.3 (14.9–15.7)	3702	13.7 (13.3–14.1)	5008	15.5 (15.1–15.9)	4989	15.7 (15.2–16.1)	4240	12.9 (12.4–13.3)	
Complete	141,871	75.3 (75.1–75.5)	18,905	69.8 (69.2–70.4)	21,049	72.7 (72.2–73.2)	21,182	76.8 (76.3–77.3)	25,113	74.8 (74.3–75.2)	24,715	75.4 (74.9–75.9)	30,907	82.3 (81.8–82.8)	
**LDL**															< 0.001^¶^
No	23,726	13.2 (13.0–13.3)	4961	18.5 (18.0–18.9)	4887	16.8 (16.3–17.2)	3519	12.7 (12.3–13.1)	4209	12.9 (12.5–13.2)	3930	12.1 (11.7–12.4)	2220	6.3 (6.0–6.5)	
Yes	162,284	86.8 (86.7–87.0)	21,899	81.5 (81.1–82.0)	24,054	83.2 (82.8–83.7)	23,993	87.3 (86.9–87.7)	29,079	87.1 (86.8–87.5)	28,686	88.0 (87.6–88.3)	34,573	93.8 (93.5–94.0)	
**LDL, mg/dL**															< 0.001^§^
< 100	73,108	44.9 (44.6–45.1)	9375	42.8 (42.1–43.5)	10,455	43.5 (42.9–44.1)	10,614	44.4 (43.8–45.0)	12,716	43.8 (43.2–44.4)	12,943	45.1 (44.5–45.7)	17,005	49.0 (48.4–49.6)	
≥ 100	89,176	55.2 (54.9–55.4)	12,524	57.2 (56.5–57.9)	13,599	56.5 (55.9–57.1)	13,379	55.6 (55.0–56.2)	16,363	56.2 (55.6–56.8)	15,743	54.9 (54.3–55.5)	17,568	51.0 (50.4–51.6)	
Mean ± SD	108.2 ± 37.6	108.3 ± 0.1	109.3 ± 36.8	109.3 ± 0.3	109.6 ± 37.5	109.5 ± 0.2	108.6 ± 37.3	108.4 ± 0.2	109.3 ± 38.0	109.2 ± 0.2	108.5 ± 37.8	108.5 ± 0.2	105.0 ± 37.9	104.9 ± 0.2	< 0.001^¶^
**Blood pressure**															< 0.001^§^
No	1033	0.6 (0.6–0.7)	269	1.1 (0.9–2.3)	398	1.41 (1.3–1.6)	214	0.8 (0.7–0.9)	62	0.2 (0.2–0.3)	24	0.1 (0.0–0.1)	66	0.2 (0.1–0.21)	
Yes	184,977	99.4 (99.4–99.4)	26,591	99.0 (98.8–99.1)	28,543	98.6 (98.4–98.7)	27,298	99.2 (99.1–99.3)	33,226	99.8 (99.8–99.9)	32,592	99.9 (99.9–100.0)	36,727	99.8 (99.8–99.9)	
**Blood pressure**															
SBP (mmHg)	130.4 ± 16.2	130.1 ± 0.0	128.8 ± 16.7	128.7 ± 0.1	128.7 ± 16.3	128.5 ± 0.1	129.0 ± 16.2	128.8 ± 0.1	129.9 ± 16.1	129.8 ± 0.1	131.7 ± 16.1	131.4 ± 0.1	133.1 ± 15.3	133.1 ± 0.1	< 0.001^¶^
DBP (mmHg)	74.4 ± 10.3	74.3 ± 0.0	74.5 ± 10.4	74.5 ± 0.1	74.4 ± 10.2	74.3 ± 0.1	74.1 ± 10.2	74.1 ± 0.1	74.1 ± 10.2	74.1 ± 0.1	74.6 ± 10.3	74.6 ± 0.1	74.6 ± 10.2	74.5 ± 0.1	< 0.001^¶^
**Controlled blood pressure**															< 0.001^§^
< 130/80 mmHg	66,425	36.5 (36.3–36.7)	10,214	38.7 (38.1–39.3)	11,175	39.7 (39.2–40.3)	10,737	39.7 (39.1–40.4)	12,423	37.6 (37.0–38.1)	10,914	33.7 (33.2–34.3)	10,962	29.8 (29.1–30.3)	
≥ 130/80 mmHg	118,552	63.5 (63.3–63.7)	16,377	61.3 (60.7–61.9)	17,368	60.3 (59.7–60.8)	16,561	60.4 (59.8–60.9)	20,803	62.4 (61.9–63.0)	21,678	66.3 (65.7–66.8)	25,765	70.2 (69.7–70.7)	

BMI: body mass index; FPG: fasting plasma glucose; HbA1c: hemoglobinA1c; LDL: low density lipoprotein cholesterol; SBP: systolic blood pressure; DBP: diastolic blood pressure.

^a^There were 7636 missing values for BMI, 1033 missing values for blood pressure.

^b^BMI was calculated as weight in kilograms divided by height in meters squared.

^§^Chi-sq.

According to the provided patient care processes and clinical outcomes, the percentage of patients with type 2 diabetes receiving a fasting plasma glucose test during their last follow-up hospital visit increased from 82.1% in 2011 to 95.1% in 2018 (*p* for trend < 0.001). We observed a decreasing trend for patients with type 2 diabetes achieving the target fasting plasma glucose level (FPG) level (70–130 mg/dL or 3.89–7.22 mmol/l) at their last follow-up visit from 40.8% in 2011 to 36.8% in 2018 (*p* for trend < 0.001) (Table 2).

### Trends in the prevalence of adequate glycemic control among patients with type 2 diabetes

**Figure 3:**
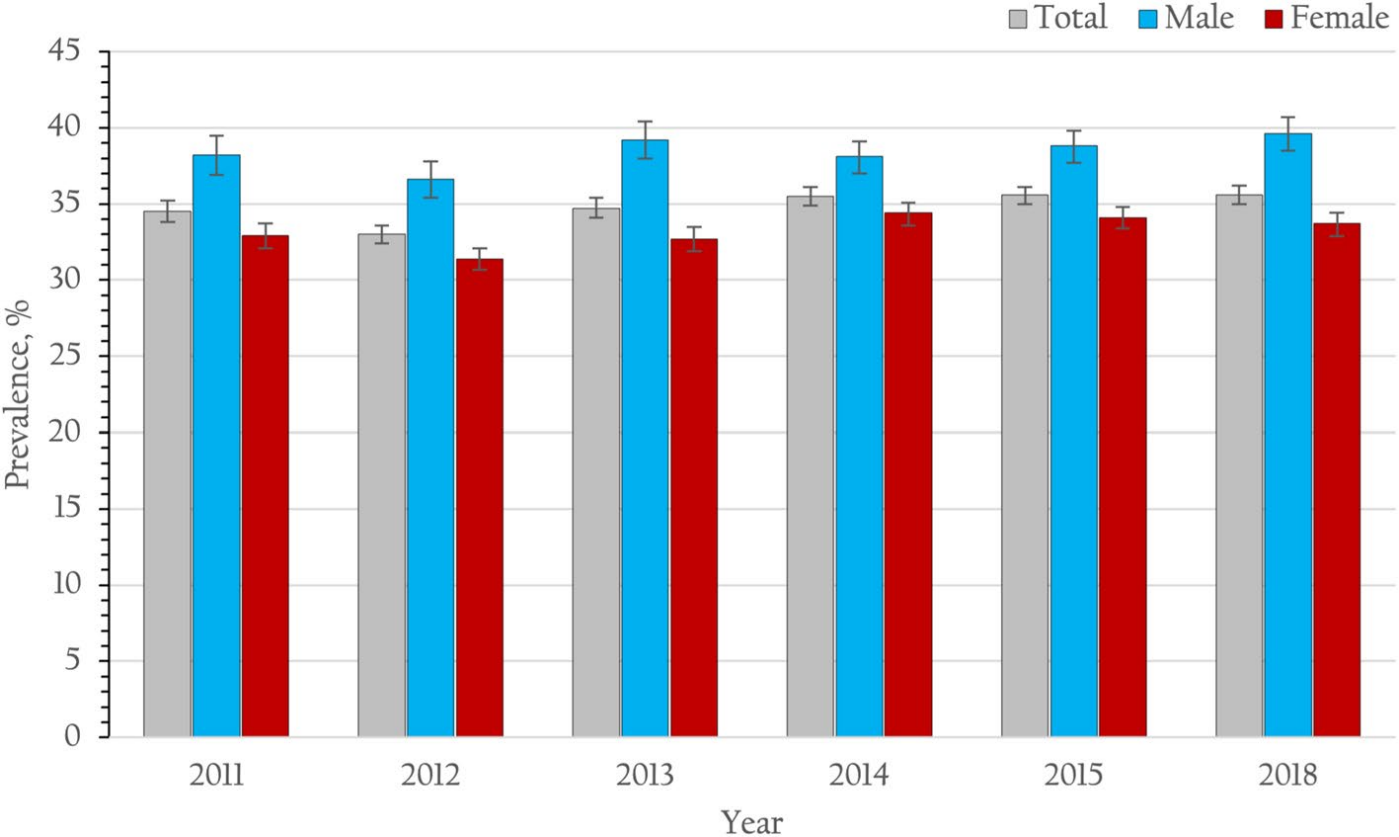
Prevalence of glycemic control (HbA1c < 7%) and 95% CI among patients with type 2 diabetes receiving continuous care in Thailand from 2011 to 2015 and 2018.

The proportion of patients receiving at least one HbA1c test during the last 12 months tended to increase (72.7, 76.2, 78.8, 77.0, 79.7, and 76.4% in 2011, 2012, 2013, 2014, 2015 and 2018, respectively), *p* for trend < 0.001. The proportion of patients presenting a HA1C level < 7% (53 mmol/mol) at their last HA1C test during the previous 12 months tended to increase (34.5, 33.0, 34.7, 35.5, 35.6 and 35.6% in 2011, 2012, 2013, 2014, 2015 and 2018, respectively), *p* for trend < 0.001 (Table 2 and Fig. 3).

### Outcomes among patients with type 2 diabetes

**Figure 4:**
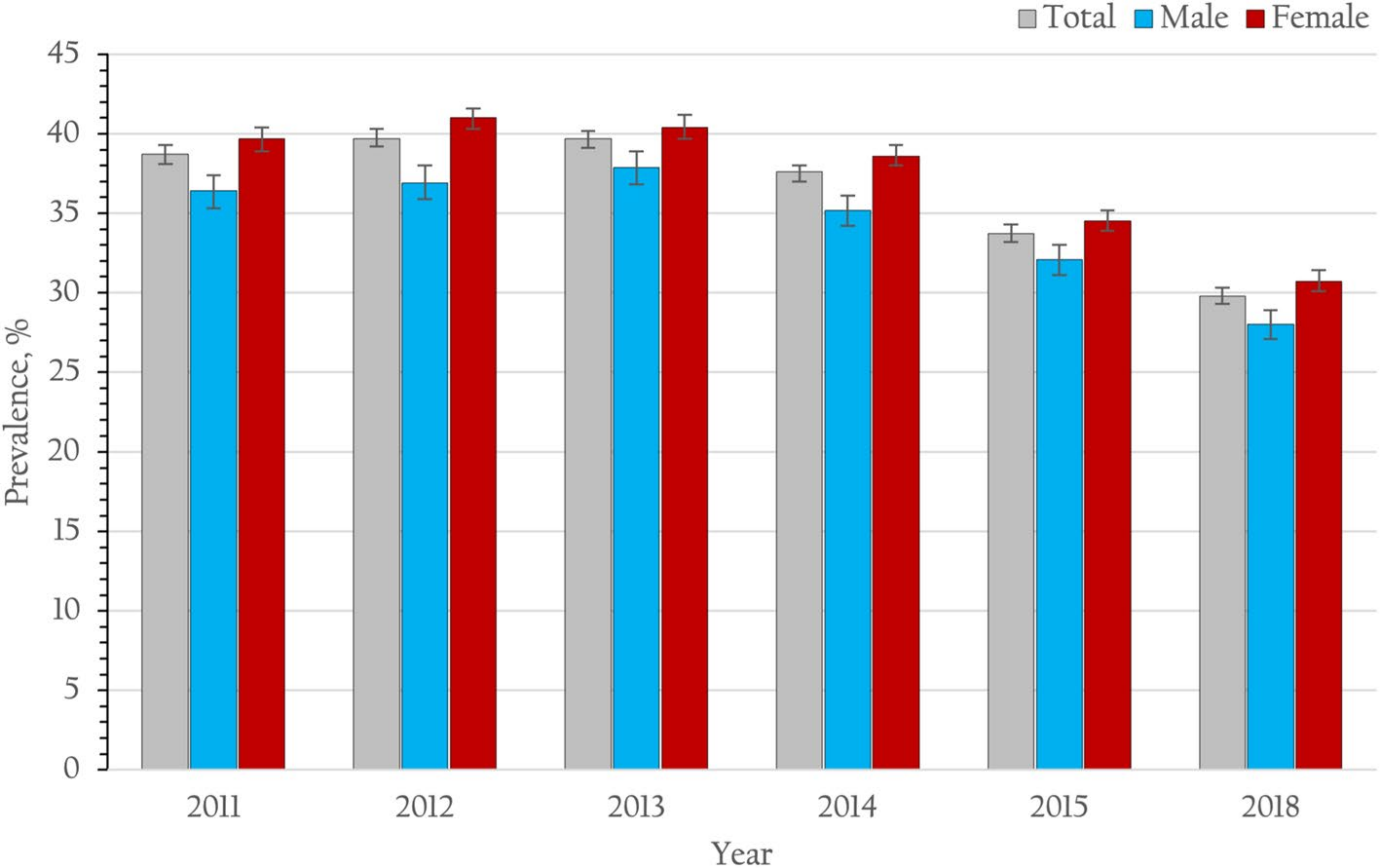
Prevalence of controlled blood pressure (< 130/80 mmHg) and 95% CI among patients with type 2 diabetes receiving continuous care in Thailand from 2011 to 2015 and 2018.

More than 80% of the patients nationwide had at least one low density lipoprotein (LDL) blood test during the previous 12 months. The prevalence of patients with diabetes achieving the target goal of an LDL level less than 100 mg/dL (2.59 mmol/l) continuously rose during the study period (from 42.8% in 2011 to 49.0% in 2018). In terms of blood pressure control, 38.7, 39.7, 39.6, 37.6, 33.7 and 29.8% of patients with type 2 diabetes achieved the blood pressure goal of less than 130/80 mmHg at the last follow-up visit in 2011, 2012, 2013, 2014, 2015 and 2018, respectively (Table 2 and Fig. 4). Additionally, 12.1, 11.0, 10.8, 10.4, 9.4 and 8.2% of patients were able to achieve the multiple goals of an HbA1c < 7% (53 mmol/mol), LDL level < 100 mg/dL (2.59 mmol/l) and blood pressure < 130/80 mmHg in 2011, 2012, 2013, 2014, 2015 and 2018, respectively. Because our data were collected nationwide, we had the opportunity to assess different patient characteristic patterns across the country. Patients type 2 diabetes from the northeast region had significantly lower BMIs (mean ± SE, 25.03 ± 0.02 kg/m^2^) than patients from other regions (mean ± SE, 25.80 ± 0.01 kg/m^2^).

### Factor associated with poor glycemic control among patients with type 2 diabetes

Regarding factors related to uncontrolled (HbA1c ≥ 7% or 53 mmol/mol) type 2 diabetes, univariate analysis revealed that age, sex, region of residence, hospital level, health insurance scheme, BMI, duration of type 2 diabetes diagnosis and comorbidity with hypertension were significantly associated with poor glycemic control (Table 3). As shown using multivariate analysis (Table 4), independent factors associated with uncontrolled type 2 diabetes (defined as patients presenting an HbA1c ≥ 7% (53 mmol/mol) at the last blood test) were assessed among all enrolled participants from 2011 to 2015 and 2018. We found that the significant independent factors for uncontrolled type 2 diabetes included being female (AOR 1.16, 95% CI 1.13–1.19), younger age (AOR 1.03, 95% CI 1.03–1.03), residing in the northeast region (AOR 1.55, 95% CI 1.51–1.61), receiving care from community hospitals (AOR 1.23, 95% CI 1.18–1.27), receiving care from general/provincial hospitals (AOR 1.21, 95% CI 1.16–1.27), underling universal health coverage scheme (AOR 1.16, 95%CI 1.12–1.20), higher BMI level (AOR1.02 , 95% CI 1.01–1.02), greater duration of type 2 diabetes (AOR 1.07, 95% CI 1.07–1.07), and not having comorbid hypertension (AOR 1.19, 95% CI 1.16–1.23).

**Table 3:** Univariate analysis for factors associated with poor achievement of glycemic control (HbA1c ≥ 7%) among patients with type 2 diabetes in Thailand from 2011 to 2015 and 2018.

Factors	Total	HbA1c > 7%	Odds ratio	95% CI	*p*-value
	n	n (%) for weighted			
**Year**					
2011	19,560	12,820 (65.4)	1.00		
2012	22,127	14,760 (67.0)	1.07	1.03–1.12	0.001
2013	21,707	14,102 (65.2)	0.99	0.95–1.03	0.692
2014	25,806	16,625 (64.4)	0.96	0.92–1.00	0.033
2015	26,307	16,771 (64.3)	0.95	0.92–0.99	0.020
2018	28,853	18,512 (64.3)	0.95	0.92–0.99	0.022
**Gender**					
Male	45,417	27,769 (61.5)	1.00		
Female	98,943	65,821 (66.8)	1.26	1.23–1.29	< 0.001
**Age, years**	60.9 ± 0.0	59.7 ± 0.0	0.97	0.97–0.97	< 0.001
< 30	279	225 (80.8)	1.00		
30–39	3164	2412 (77.3)	0.81	0.58–1.12	0.198
40–49	17,435	12,853 (73.8)	0.67	0.49–0.92	0.013
50–59	43,059	30,226 (70.3)	0.56	0.41–0.77	< 0.001
60–69	47,991	30,567 (63.9)	0.42	0.31–0.58	< 0.001
70–79	26,184	14,381 (55.2)	0.29	0.21–0.40	< 0.001
≥ 80	6248	2926 (46.7)	0.21	0.15–0.29	< 0.001
**Regions**					
Central	48,527	29,171 (60.1)	1.00		
North	29,495	18,703 (63.7)	1.16	1.13–1.20	< 0.001
Northeast	44,977	32,166 (71.2)	1.64	1.60–1.69	< 0.001
South	21,361	13,550 (62.7)	1.12	1.08–1.16	< 0.001
**Hospital level**					
Regional hospital	17,208	10,639 (62.1)	1.00		
General hospital	33,455	21,151 (63.0)	1.04	1.00–1.08	0.051
Community hospital	93,697	61,800 (66.4)	1.21	1.16–1.25	< 0.001
**Scheme**					
Government officer	25,967	15,448 (59.5)	1.00		
Universal health coverage	106,931	70,809 (66.6)	1.36	1.32–1.40	< 0.001
Social security	5908	3905 (66.2)	1.33	1.25–1.42	< 0.001
Others	5554	3428 (61.7)	1.10	1.03–1.17	0.003
**BMI, kg/m**^**2**^	25.5 ± 0.0	25.7 ± 0.0	1.02	1.02–1.02	< 0.001
18.5–22.9	34,875	21,694 (62.7)	1.00		
< 18.5	4907	2919 (59.9)	0.89	0.83–0.95	< 0.001
23.0–24.9	27,720	17,954 (65.1)	1.11	1.07–1.15	< 0.001
25.0–29.9	50,330	33,202 (66.9)	1.20	1.17–1.24	< 0.001
≥ 30	21,072	14,258 (67.8)	1.25	1.21–1.30	< 0.001
**Diabetes duration, years**	7.3 ± 0.0	7.7 ± 0.0	1.05	1.04–1.05	< 0.001
≤ 6^a^	69,216	41,890 (60.5)	1.00		
> 6	69,363	47,904 (69.1)	1.46	1.42–1.49	< 0.001
**HT comorbidity**					
Yes	108,267	68,077 (72.7)	1.00		
No	36,093	25,506 (27.3)	1.41	1.38–1.45	< 0.001

BMI; body mass index, HT; hypertension, ^a^cut-off points at the median, CI; confidence interval.

**Table 4:** Multivariate analysis for factors associated with poor achievement of glycemic control (HbA1c ≥ 7%) among patients with type 2 diabetes in Thailand from 2011 to 2015 and 2018.

Factors	Adjusted OR	95% CI	*p*-value
**Year**			
2011	1.00		
2012	1.05	1.00–1.10	0.040
2013	0.99	0.94–1.03	0.609
2014	0.94	0.90–0.97	0.011
2015	0.92	0.88–0.96	< 0.001
2018	0.94	0.90–0.99	0.011
**Gender**			
Male	1.00		
Female	1.16	1.13–1.19	< 0.001
**Age, years**	0.97	0.97–0.97	< 0.001
**Regions**			
Central	1.00		
North	1.10	1.06–1.14	< 0.001
Northeast	1.55	1.51–1.60	< 0.001
South	1.10	1.06–1.14	< 0.001
**Hospital level**			
Regional hospital	1.00		
General hospital	1.21	1.16–1.27	< 0.001
Community hospital	1.23	1.18–1.27	< 0.001
**Scheme**			
Government officer	1.00		
Universal health coverage	1.16	1.12–1.20	< 0.001
Social security	1.00	0.94–1.07	0.971
Others	1.04	0.97–1.11	0.258
**BMI, kg/m**^**2**^	1.02	1.01–1.02	< 0.001
**Diabetes duration, years**	1.07	1.07–1.07	< 0.001
**HT comorbidity**			
Yes	1.00		
No	1.19	1.16–1.23	< 0.001

BMI: body mass index; HT: hypertension; OR: odds ratio; CI: confidence interval.

## Discussion

We successfully enrolled 186,010 patients with type 2 diabetes receiving care at public hospitals and clinics from 2011 to 2015 and 2018 nationwide. This study constituted the largest epidemiological study among patients with type 2 diabetes in Thailand to date. We found that one third of patients with type 2 diabetes receiving continuous care in Thailand maintained adequate glycemic control (HbA1c < 7% or 53 mmol/mol). Because our study population was selected from those patients attending the clinic on the enrollment date the outcomes from our study may represent only patients who were relatively engaged in care.

Approximately two thirds of the enrolled participants with type 2 diabetes were female. However, data from a National Health Examination Survey in Thailand in 2014 (the NHES V) showed that the prevalence rates of type 2 diabetes among people older than 20 years were relatively comparable, with prevalence rates of 8.9% (95%CI 8.3–9.5) and 10.8% (95%CI 10.2–11.4) among males and females, respectively. Nevertheless, the percentage of undiagnosed type 2 diabetes was 51.2% (95%CI 45.9–56.6) and 41.3% (95%CI 36.6–46.1) among males and female patients with type 2 diabetes in Thailand, respectively^4^. This phenomenon of sex difference in type 2 diabetes care may be in part explained by gender values and health seeking behavior in that male patients exhibited lower levels of health awareness including seeking out and engaging in diabetes self-management behaviors^12,13^. The higher ratio of female patients with type 2 diabetes receiving diabetes care in hospitals compared with the ratio found in the community may reflect the lower accessibility of care among male patients with type 2 diabetes in Thailand. This finding indicates that specific programs aimed at increasing early detection and accessibility to continuous care among male patients with diabetes require further attention in Thailand.

The current universal health care policy in Thailand comprised 3 major health care schemes: the civil servant medical benefit scheme (for all civil servants and their immediate family members, ~ 5 million people), the social security scheme (for private employees, ~ 10 million people) and the universal health coverage scheme for the rest of the Thai population (~ 50 million people). All of these schemes provide free medical care for patients, including patients with type 2 diabetes. Our study found that patients with type 2 diabetes under the civil servant medical benefit scheme had a lower proportion of uncontrolled (HbA1c ≥ 7% or 53 mmol/mol) type 2 diabetes (59.57%) than patients under the universal health coverage scheme (66.65%). However, these proportions did not differ significantly from patients under the social security scheme (66.23%). These findings suggested that an opportunity to improve the quality of type 2 diabetes care exists in the universal health coverage and social security schemes.

We also found a difference in diabetes control among hospital levels. Patients with type 2 diabetes from regional hospitals had a lower proportion of uncontrolled diabetes (HbA1c ≥ 7% or 53 mmol/mol) than that of patients from general and community hospitals. One explanation for this finding is that the more comprehensive care provided by specialists in regional hospitals may produce better clinical outcomes.

The proportion of patients receiving a blood test for fasting plasma glucose, HbA1c, and LDL increased annually, which might indicate an increase in care quality. The mean HbA1C levels among the enrolled patients with type 2 diabetes were 8.03% (64.3 mmol/mol) in 2011 and slightly decreased to 7.97% (63.6 mmol/mol) in 2018. The mean HbA1C level found in this study was comparable to that in a study conducted in Malaysia during the same period^14^. A total of 34.47% and 35.59% of the patients in this study achieved the target HbA1c of less than 7% (53 mmol/mol) in 2011 and 2018, respectively, which was also comparable to the data from Asian countries including Malaysia^14^, Saudi Arabia^15^ and China^16^. However, the percentages of controlled patients with diabetes from the present study were lower than those in the reported data from the US from 1999 to 2010 (52.2%)^17^.

Women in this study were more likely to have poor glycemic control than male patients, which was consistent with other reports^18–21^. One explanation for this sex difference in glycemic control may be related to biological factors, including the effects of female sex hormones on the action of insulin^22^. Additionally, the observed differences may be due to differences in health care behavior, including lower treatment adherence among women^23^.

In our study, younger patients with type 2 diabetes exhibited poorer glycemic control than older patients, which was consistent with findings from other settings, including the US^17^. Older patients may be more motivated to take care of their diabetes and more compliant with their medication adherence and healthy eating behavior^24^. Younger patients may be more likely to disregard diabetes as important and be less adherent to medication, lifestyle modifications and diet restrictions^25^. A related study found that the persistence of HbA1c elevation among younger individuals could be due to an inadequate low medication dosage or the infrequent use of combined drug regimens^26^. Additionally, younger patients are also less likely to have an established diagnosis of hypertension which might have mediated the observed association between poorer glycemic control and the absence of a diagnosis of clinical hypertension. Therefore, younger patients with type 2 diabetes should constitute a target population to improve glycemic control and other diabetes care. More specific programs customized for this target working-age population should be implemented in Thailand. An effective program for younger patients would be beneficial and decrease the burden of diabetes complications in the near future.

This study found that patients with type 2 diabetes from the northeast region had a lower percentage of adequate glycemic control than patients from other regions. Factors related to the health system and personal factors may play roles in the poor glycemic control among patients from the northeast. The northeast region has the lowest physician-to-population ratio relative to the rest of the country^27^. The relatively limited health care resources in this region might have influenced the outcome of diabetes care. In terms of personal factors, glutinous rice is a staple food item in this part of the country. Glutinous rice has a high carbohydrate content, high glycemic index and a high risk of hypertriglyceridemia^28^ compared with nonglutinous rice, which is the staple food of the other regions.

We found that patients with type 2 diabetes with greater BMI level tended to be at risk for poor glycemic control. Similarly, one related study in the US reported positive associations between being overweight or obese and having uncontrolled type 2 diabetes^29^. Additionally, strong evidence was found that obesity management could be beneficial in treating type 2 diabetes^30,31^. Related studies have reported that the sustained weight loss has been illustrated to improve glycemic control^32,33^. Thus, the dietary therapy and weight management should be encouraged for patients with type 2 diabetes to better glycemic control.

One limitation of this study was the representativeness of the study subject. The study included only patients with type 2 diabetes visiting a hospital for diabetes care and did not include patients with type 2 diabetes receiving care at primary care units, accounting for approximately one half of the overall patients with type 2 diabetes in Thailand in 2015. Additionally, the study did not include subjects from university hospitals. Because this study was the first endeavor to examine type 2 diabetes care outcomes systematically, nationwide in Thailand, we did not have an opportunity to compare the outcomes with the period before the implementation of universal health care coverage in 2002. Limitations of the validity related to factors associated with uncontrolled type 2 diabetes may have occurred in this study. Because the HbA1c blood test was used to define uncontrolled patients with type 2 diabetes (HbA1C > 7% or 53 mmol/mol), the analysis was limited to the group of patients tested for HbA1c during the previous 12 months (72.70, 76.23, 78.80, 77.00, 79.69 and 76.39 in 2011, 2012, 2013, 2014, 2015 and 2018, respectively). Patients not receiving HbA1C tests were not randomized and were more likely to be under the universal health coverage scheme, visit community hospitals, have a shorter duration of diabetes diagnosis and reside in the northern region of the country. Moreover, we only collected data from May to August of each year between 2011 and 2018, although glycemic control may vary by season^34–37^.

## Conclusion

In conclusion, the study examined the current standards of diabetes care nationwide in hospitals in Thailand. The results showed room for further improvement in the quality of diabetes care. The study findings are useful for health care providers comparing performance and planning quality improvement initiatives. Variations in the outcome of diabetes care were observed among patients of different ages, sex, durations of diabetes, and hypertension comorbidity status and among different hospital levels. At the policy level, the pay-for-performance and chronic disease management programs discussed earlier may improve the current situation^38^. At the hospital level, regular educational activities, practice guideline development and clinical audits are useful for continuous quality improvement.

## Supplementary Information

Supplementary Information.

## Data Availability

Data cannot be shared publicly because the data set contains identifying information; additionally, the data belong to the Thailand DM/HT study of the Medical Research Network of the Consortium of Thai Medical Schools (MedResNet). Thus, ethical restrictions exist on the data set. Data are available from the Thai National Health Security Office (NHSO), Bangkok, Thailand (contact via sirikorn@nhso.go.th) for researchers who meet the criteria for access to confidential data.

## Abbreviations

HbA1c: HemoglobinA1c
LDL: Low-density lipoprotein
NHSO: National Health Security Office
MoPH: Ministry of Public Health
MedResNet: Medical Research Network of the Consortium of Thai Medical Schools
BMI: Body mass index
Adj. OR: Adjusted odds ratio
95% CI: 95% confidence interval
