# Supplementary material for: National trends in the prevalence of glycemic control among patients with type 2 diabetes receiving continuous care in Thailand from 2011 to 2018
Source: Sci Rep. 2021 Jul 12;11:14260. doi: 10.1038/s41598-021-93733-4 (PMC8275592; doi:10.1038/s41598-021-93733-4)
Supplement: Supplementary file 1 — Supplementary Information. [file 41598_2021_93733_MOESM1_ESM.pdf]

An Assessment on Quality of Care among Patients Diagnosed with Type 2 Diabetes and Hypertension Visiting the Ministry of Public Health (MoPH) and Bangkok Metropolitan Administration Hospitals in Thailand (Thailand DM/HT)

Hospital Code : ☐☐☐☐☐

PID : ☐☐☐☐

Date : ☐☐ / ☐☐ / ☐☐

Type 2 diabetes was diagnosed since: ☐☐☐☐, ☐ **Not recorded**

Part 1: Demographic characteristics

1. Sex ☐ 1. Male ☐ 2. Female

2. Age ☐☐☐ years (Current year – Birth year)

3. Occupation (Current)

☐ 1. Non occupation
☐ 2. Priest
☐ 3. Students
☐ 4. Government officer
☐ 5. Government employee
☐ 6. State enterprise employee
☐ 7. Private sector employee
☐ 8. Private business
☐ 9. Agriculturist
☐ 10. Fisherman
☐ 11. Daily hiring
☐ 12. Others……………………………
☐ 13. Not recorded

4. Religions

☐ 1. Buddhist ☐ 2. Islamic ☐ 3. Christian
☐ 4. Others……………………………

5.Scheme

☐ 1. Universal health coverage scheme
☐ 2. Government officer
☐ 3. Social security scheme
☐ 4. State enterprise scheme
☐ 5. Cash
☐ 6. Others………………………………………
☐ 7. Not recorded

6. Last body weight (in 12 months) ☐☐☐.☐ kg. ☐ **Not recorded**

7. Last height (in 12 months) ☐☐☐.☐ cm. ☐ **Not recorded**

8. Last waist circumference (in 12 months) ☐☐☐.☐ cm. ☐ **Not recorded**

Hospital Code: ☐☐☐☐☐ **PID:** ☐☐☐☐

Part 2: History of smoking and alcohol drinking

9. Smoking status

☐ 1. Current smoker ☐ 2. Ex-smoker ☐ 3. Never ☐ 4. Not recorded

10. Received the recommendation for smoking cessation (last 12 months)

☐ 1. No

☐ 2. Yes, on ☐☐/☐☐/☐☐☐☐

By ☐ 1. Doctor/Nurse ☐ 2. Psychologist/Social worker

☐ 3. Trained staff ☐ 4. Not known

☐ 3. Not recorded

11. Alcohol drinking

☐ 1. Current smoker ☐ 2. Never ☐ 3. Ex-drinker ☐ 4. Not recorded

Frequency

☐ 1.1 Daily ☐ 1.2 3-4 times/week ☐ 1.3 1-2 times/week

☐ 1.4 1-2 times/months ☐ 1.4 Occasionally

Part 3 Comorbidities

DIAGNOSES	NO	YES	DATE OF DIAGNOSIS
**13. Hypertension**	☐	☐	☐☐/☐☐/☐☐☐
**14. Dyslipidemia**	☐	☐	☐☐/☐☐/☐☐☐
**15. Gout**	☐	☐	☐☐/☐☐/☐☐☐
**16. Renal insufficiency** (CRI, CRF, CKD, ESRD)	☐	☐	☐☐/☐☐/☐☐☐
**17. Microalbuminuria**	☐	☐	☐☐/☐☐/☐☐☐
**18. Macroalbuminuria**	☐	☐	☐☐/☐☐/☐☐☐
**19. Diabetic kidney disease; DKD**	☐	☐	☐☐/☐☐/☐☐☐
**20. Diabetic retinopathy; DR**	☐	☐	☐☐/☐☐/☐☐☐
**21. Others**..	☐	☐	☐☐/☐☐/☐☐☐

Part 4 History of medication used (last visit)

22. Anti-hyperglycemic medication ☐ 1. No ☐ 2. Yes

☐ 1. Biguanides ☐ 2. Sulfonylurea ☐ 3. Non-sulfonylurea

☐ 4. Thiazolidinedione ☐ 5. Alpha-glucosidase Inhibitor

☐ 6. DPP-4 Inhibitor ☐ 7. GLP-1 Analog ☐ 8. Insulin

☐ 9. SGLT2 inhibitor ☐ 10. Others..

23. Anti-hypertensive medication ☐ 1. No ☐ 2. Yes

☐ 1. Diuretics ☐ 2. Beta-blocker

☐ 3. Alpha-blocker ☐ 4. Calcium channel blockers

☐ 5. Angiotensin converting enzyme inhibitors (ACEI)

☐ 6. Angiotensin receptor blockers (ARB) ☐ 7. Vasodilators

☐ 8. Central agonists

☐ 9. Peripheral adrenergic inhibitors

☐ 10. Others..

Hospital Code: ☐☐☐☐☐ **PID:** ☐☐☐☐

Part 5 History of medication used (last 12 months)

24. Using ACEI, ARB in last 12 months

☐ 1. No ☐ 2. Yes (in last 12 months)

Drugs	Currently used	Give up in last 12 months	Date (give-up)
☐ 1.ACEI	☐	☐	☐☐/☐☐/☐☐☐☐
☐ 2.ARB	☐	☐	☐☐/☐☐/☐☐☐☐

25. History of antiplatelet and anticoagulant medication used

☐ 1. No ☐ 2. Yes (in last 12 months)

☐ 1. Aspirin 81 mg

☐ 2. Clopidogrel (Pidogen, Apolets, Ceruvin)

☐ 3. Prasugrel (Effient)

☐ 4. Ticlopidine (Aplaket, Cenpidine, Ticlid, Ticlo, Ticlodin, Ticlopine, Tilopin, Tipidine)

☐ 5. Others……………………………………………………

Part 6 Blood pressure level (last 12 months)

No.	Date	Blood pressure (mmHg)
**26.** Last visit	☐☐/☐☐/☐☐☐☐	☐☐☐/☐☐☐
**27.** Before last visit	☐☐/☐☐/☐☐☐☐	☐☐☐/☐☐☐

Part 7 Laboratory testing

Test	No	Yes	Results	Unit	Date
**28. FPG (last)**	☐	☐	☐☐☐	mg/dL	☐☐/☐☐/☐☐☐☐
**29. FPG (before last)**	☐	☐	☐☐☐	mg/dL	☐☐/☐☐/☐☐☐☐
**30. HbA1c (last)**	☐	☐	☐☐.☐	%	☐☐/☐☐/☐☐☐☐
**31. HbA1c (before last)**	☐	☐	☐☐.☐	%	☐☐/☐☐/☐☐☐☐
**32. Hct**	☐	☐	☐☐.☐	%	☐☐/☐☐/☐☐☐☐
**33. Hb**	☐	☐	☐☐.☐	g/dL	☐☐/☐☐/☐☐☐☐
**34. Serum BUN**	☐	☐	☐☐.☐	mg/dL	☐☐/☐☐/☐☐☐☐
**35. Serum Creatinine (last)**	☐	☐	☐☐.☐	mg/dL	☐☐/☐☐/☐☐☐☐
**36. Estimate GFR (last)**	☐	☐	☐☐☐.☐	ml/min/ 1.73m²	☐☐/☐☐/☐☐☐☐
**37. Serum Potassium**	☐	☐	☐☐.☐	mmol/L	☐☐/☐☐/☐☐☐☐
**38. Serum Uric Acid**	☐	☐	☐☐.☐	mg/dL	☐☐/☐☐/☐☐☐☐
**39. Total Cholesterol**	☐	☐	☐☐☐.☐	mg/dL	☐☐/☐☐/☐☐☐☐
**40. Triglyceride**	☐	☐	☐☐☐.☐	mg/dL	☐☐/☐☐/☐☐☐☐
**41. HDL Cholesterol**	☐	☐	☐☐☐.☐	mg/dL	☐☐/☐☐/☐☐☐☐
**42. LDL Cholesterol**	☐	☐	☐☐☐.☐	mg/dL	☐☐/☐☐/☐☐☐☐
